# Self-employment convergence in Europe: The role of migration

**DOI:** 10.1371/journal.pone.0250182

**Published:** 2021-04-22

**Authors:** Ana Cuadros, Juan Carlos Cuestas, Joan Martín-Montaner

**Affiliations:** 1 Department of Economics and IEI, Universitat Jaume I, Castellon, Spain; 2 Department of Economics and Finance, Tallinn University of Technology, Tallinn, Estonia; TED University, TURKEY

## Abstract

This paper attempts to identify patterns of convergence in the rates of self-employment (SE) for both foreign-born and natives in a sample of 17 European countries during the period 1999–2018. A distinction is made between self-employed with and without workers. Our analysis is relevant for policy-making: whether or not there is evidence of convergence in SE rates can be an indication of the homogeneity of self-employed workers among the countries analysed, which in turn may reflect the success of the EU-wide policies to boost SE.

## 1. Introduction

Entrepreneurship is often pointed to as an important contributor to economic growth, job creation, innovation and production efficiency [1–3]. It is associated with more job creation compared to the effects of existing or new firms hiring salaried employees [4] It also has a positive effect on labour market integration as well as competition due to an increase in the number of businesses [5].

This evidence explains why policy-makers are paying increasing attention to the function fulfilled by entrepreneurship in generating new jobs and investments. The European Commission (EC) has acknowledged in its Europe 2020 strategy that self-employment (SE) and entrepreneurship have a positive impact on economic growth and job creation. Thus, a series of projects such as the European Progress Microfinance Facility, the Employment and Social Innovation Programme and the European Social Fund have been created to promote entrepreneurship (The Entrepreneurship 2020 Action Plan, Internal Market, Industry, Entrepreneurship and SMEs)The implementation and subsequent evaluation of the effectiveness of these programmes, however, present several difficulties.

Firstly, measuring entrepreneurship is a difficult task. As it is a behavioural characteristic, we can only use indirect indicators. The concept (and measurement) of entrepreneurship has usually been linked to SE, but there are important differences [6]. On the one hand, individuals may display entrepreneurial attitudes while working as employees ([1] label such people ‘intrapreneurs’). On the other hand, not all people in SE are motivated entrepreneurs pursuing a business opportunity. There are also individuals that become self-employed out of necessity, being pushed into starting a business because they have no other employment options. Obviously, it is the opportunity self-employed individuals that we usually consider to be entrepreneurs, and it is they who create more jobs and enhance regional economic growth and prosperity [7, 8]. (It is worth recalling that the distinction between opportunity and necessity entrepreneurship refers to the motivation for starting a business. People can build high-growth, job-creating companies even if their motivation for starting a business was necessity (see [9]).

Secondly, the implementation of policies such as those designed by the European Commission are heterogeneous in scope, as SE patterns vary across countries and segments of population. The Global Entrepreneurship Monitor (GEM) shows that entrepreneurship activity is more prevalent in countries with greater income inequality, while various studies reveal the relevance of local institutions and conditions in determining SE rates ([10] or [11], among others). Generally, European countries display higher self-employment rates than other OECD countries: In 2019, the SE rate in the Euro Area was 14.7%, considerably higher than in the US (6.3%), Canada (8.3%) or Australia (9.6%). (OECD (2020), Self-employment rate (indicator). doi: 10.1787/fb58715e-en). However, the Euro Area itself is not a homogeneous region: rates range from values below 10%, such as those in Denmark (8.3%) or Germany (9.6%), to values above 20%, such as in Italy (22.7%) or Greece (31.9%). This diversity could undermine the impact of the European Union’s commitment to promoting entrepreneurship.

In this context, the analysis of convergence in SE patterns may provide evidence that can contribute to a better design of public policies. However, this literature is still scarce [12] find evidence of weak convergence across Europe, although conditional convergence is observed in both Southern and Northern Europe. Countries in Southern Europe share several social and institutional characteristics and economic structures, as do countries in Northern Europe. Thus, the identification of these two clubs of convergence should not come as a surprise. However, differences in SE across countries are not only associated with the structure of national economies but also, with the characteristics of the national population, as [13] observe for gender issues. These authors analyse whether there is SE rate convergence between genders, providing evidence of the difference between core and peripheral countries in Europe (See [11] for the analysis of institutional features as determinants of national differences in SE rates).

To the best of our knowledge, no analysis of convergence in SE carried out so far has taken into account individuals’ country of birth. Due to the specific issues concerning migrant SE, the pattern of convergence (if any) in their SE rates may not necessarily be the same as that of natives. Although we might assume that foreign-born individuals who are self-employed face the same institutional frictions as natives do, they may also face potential obstacles to joining their host countries’ labour markets that make them more likely than natives to enter SE. It is thus worth checking whether different patterns can be observed in most of the receiving countries. If all migrants face similar problems across countries, it could lead to stronger convergence than for natives. However, less convergence could be expected if migrants from different cultures have different attitudes towards SE.

How does migration affect entrepreneurship and SE? Since [14], a greater propensity to SE has generally been attributed to the migrant population. However, evidence of a systematic and increasing SE rate for the migrant population mainly comes from the United States [5, 15, 16]. According to [17, 18], first-generation immigrants create about 25 percent of all new businesses in the United States; this percentage jumps to 40 percent in some heavily immigrant-populated states like California and New York.

Migrant SE in European countries constitutes a different experience. Unlike in the United States, migrants are less likely than natives to be self-employed in about half of the OECD countries [19], and this is especially true in the countries with larger immigrant populations, such as Germany, Italy, Spain, Switzerland and the Netherlands [20]. Thus, although total SE is relatively higher in Europe than in other OECD countries, migrant SE is lower. In light of this evidence, the EC incorporated the promotion of migrant SE into its strategy for 2020, as one of the keys to "smart, sustainable and inclusive growth”. It was an important decision as migrant SE is arguably responsive to public policies [21]. The key question, of course, is whether a homogeneous response to this policy is to be expected for all foreign-born self-employed workers in all countries. As is the case with native SE, there are strong asymmetries between Northern and Southern countries in the share of foreign-born citizens entering SE. The different migration history in the two regions is likely to explain most of these differences [22].

This paper focuses on the analysis of convergence of SE rates in Europe for native and migrant populations, considering different types of SE (employers and own-account workers). We observe that foreign-born SE rates for individual countries always converge to country averages, which are different for each type of SE. However, the evidence obtained for convergence of native SE rates is weak in all cases. These outcomes suggest that migrant self-employed workers contribute to the homogenization of SE rates across countries. The relevance of our findings for policy-making decisions is straightforward: The fact that non-native self-employed behave similarly across countries makes EU policies easier to implement and supports the design of EU-wide policies to boost SE.

The rest of the paper is organised as follows. Section 2 summarises previous studies reporting an above-average SE rate for migrants as well as its main explanatory factors. Section 3 describes the data. Section 4 explains the empirical approach and presents the main results. We first analyse the existence of convergence in SE rates among a group of EU countries for both the native and the foreign-born population. Second, in order to identify the prevalence of opportunity versus necessity SE, we distinguish between employers (self-employed workers who are job creators) versus own-account workers (self-employed without employees). Conclusions and policy recommendations are presented in the last section.

## 2. Background. Is there a self-employment premium for migrants?

As previously mentioned, empirical research has systematically observed different SE patterns between native and migrant populations. The debate, however, centres on the causes behind this different behaviour. Three main explanations have been suggested. The first is related to migrants’ poor integration into the labour markets of the recipient countries (or clear discrimination against them), which could prevent them from accessing jobs and push them to seek SE. As stated by [5], SE as a labour market stepping stone in the host country is most relevant among lower-skilled migrants. Difficulties associated with a lack of knowledge of the local language or local customs in host country labour markets could leave migrants with no choice but to create their own business. However, whenever they have a choice, migrants may prefer wage employment to being self-employed. These authors also find that there is no strong evidence that SE is an effective tool of upward economic mobility among low-skilled immigrants.

The second explanation refers to a process of self-selection of migrants: individuals who are willing to move to a foreign country may be positively self-selected for entrepreneurial mindsets and interest. Since international mobility and entrepreneurial action are both characterised by higher risks and (presumably) higher returns than employment in the domestic labour market, individuals with a higher propensity for risky actions might be more inclined to engage in these behaviours [16]. In other words, in their decision to migrate, migrants are showing that they may be less risk averse [23]. Rather than being a consequence of the individual’s innate entrepreneurial nature [16], attribute a higher entrepreneurial activity of migrants to their extensive cross-cultural experience, which may improve their ability to identify business opportunities. Their higher exposure to different cultures may allow the transfer of knowledge about customer problems or solutions from one country to another [24] demonstrate that cultural diversity breeds entrepreneurship, although recent migrants (rather than the descendants of past migrants) are those who create the conditions for a more dynamic entrepreneurial environment. Similarly [25], claim that “… significant time commitment, the acceptance of deferred gratifications and higher risks …” are characteristics of job creators.

Finally, it has also been argued that there could be a kind of SE culture transferred from migrants’ country of origin to their host country. According to this line of argument, in migrants’ country of origin (usually lower-income countries than their destination countries) people would show a greater propensity to start their own business, mainly because of the less developed business environment, which would hinder the possibility of becoming a salaried employee. However, the evidence for this hypothesis is mixed [26]. [27] and [28] find a positive link, but the strength of the entrepreneurial culture is different across ethnicities [29] observe differences in SE rates between ethnic and racial groups and do not support the "culture" argument, since SE rates in host countries are not related to SE rates in their countries of origin. The proportion of SE in migrants’ countries of origin could be more related to the development stage of those countries than to a cultural attitude towards SE (see [30] or [31]).

Authors of some recent studies are also sceptical about the view of migrants as more likely to be self-employed. According to [32], the existence of a migrants’ SE premium could be explained by the fact that migrants often tend to be located at the extremes of the ability distribution (that is, those positions located either at the very top or at the bottom of the wage distribution). These authors highlight that both “stars” (individuals at the very top of the wage distribution) as well as “misfits” (those at the bottom) are more likely to become self-employed. Besides, although rates of business ownership are higher among migrants, promoting SE is unlikely to improve outcomes for the less skilled.

In similar terms, [19] criticise the general view of migrants as super-entrepreneurs. They stress that both migrants and non-migrants would be more or less likely to be self-employed as a result of their individual characteristics. According to these authors, a substantial number of self-employed migrants do not create jobs, most do not innovate and a substantial number of migrants’ start-ups disappear after a few years. In other words, the challenges faced by migrant self-employed are not significantly different from those faced by non-migrants (apart from discrimination, which may foster SE).

One important implication stemming from the above-mentioned research is that migrants’ greater integration in the receiving countries (meaning they are less likely to be underpaid in established firms) should lead to a reduction only in necessity SE. We should not expect the same trend in the case of opportunity SE, which is more akin to the vision of migrants as super-entrepreneurs. The above considerations are relevant for the empirical research carried out in the present paper. Research on the convergence in SE rates has been gathering momentum, as it is a good way to assess real convergence and whether the EC policies are having an impact on SE rates [21].

Thus, convergence analysis can contribute to our understanding of the different patterns of migrants’ and natives’ SE in different countries. But gaining such an understanding requires us to consider the different personal and institutional characteristics that lead to SE in both groups. We assume that discrimination or poor integration into labour markets in the receiving countries could be more closely linked to necessity SE, whereas SE stemming from self-selection by migrants is likely to be associated with opportunity SE. Given that we lack individual data to examine whether individuals’ SE decision is driven by opportunity or necessity, we proxy those decisions by distinguishing between those who are able to hire other people (employers) and those who remain self-employed without employees (own-account workers). In the next section, we describe the profile of SE in the native and foreign-born populations of the countries in our sample, considering this distinction. Following this descriptive section, we present our convergence analysis.

## 3. Data description

The data for the empirical analysis consists of series of the number of self-employed people per country, both native and foreign-born, normalised by the active population of both groups. We also distinguish between employers and own-account workers for both native and foreign-born (15–64 years). The data come from *Eurostat* (code *lfsa_esgacob*) in annual frequency for 17 EU countries; namely, Austria, Belgium, Cyprus, Denmark, Estonia, Finland, France, Germany, Greece, Ireland, Luxembourg, the Netherlands, Norway, Portugal, Spain, Sweden and the United Kingdom. The data span the period 1999–2018. The countries have been selected according to the availability of the data. Estonia has been dropped in the case of employers and own-account workers statistics and Finland in the case of employers because of the lack of data.

**Fig 1:**
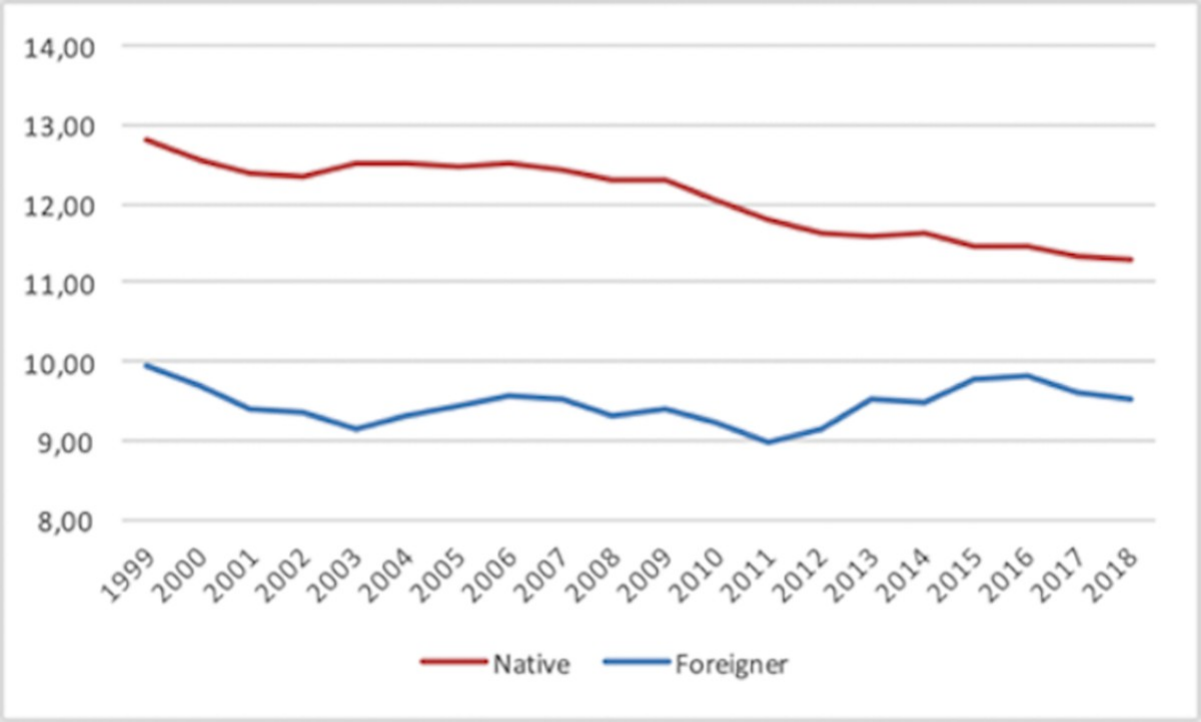
Total self-employment rate 1999–2018. Country average.

**Fig 2:**
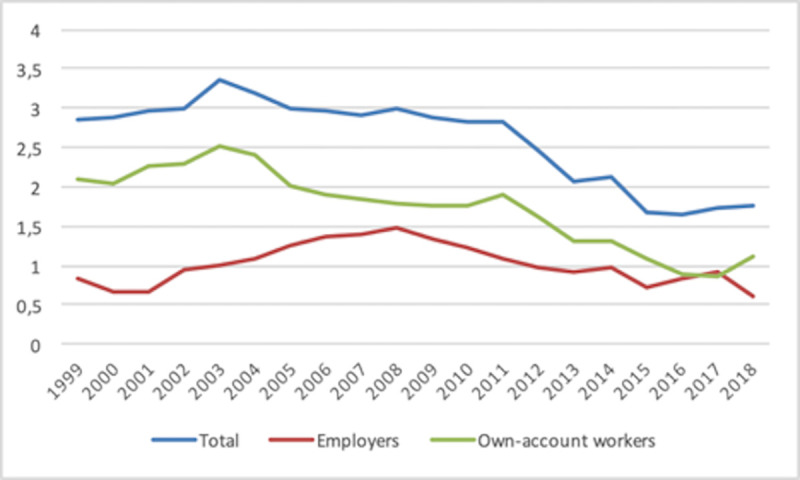
Native vs. foreign-born self-employment rate differences. 1999–2018. Country average.

Regarding the total SE rate (Fig 1), we observe that the rate for native individuals is always higher than the rate for foreign-born individuals (consistent with other evidence mentioned in the Introduction). The former displays a steady downward trend, whereas the latter is more or less stable over time (even moving slightly upwards in the 2011–2015 period). As a consequence, both rates steadily approach one another until 2016. This pattern is clearer in Fig 2.

**Fig 3:**
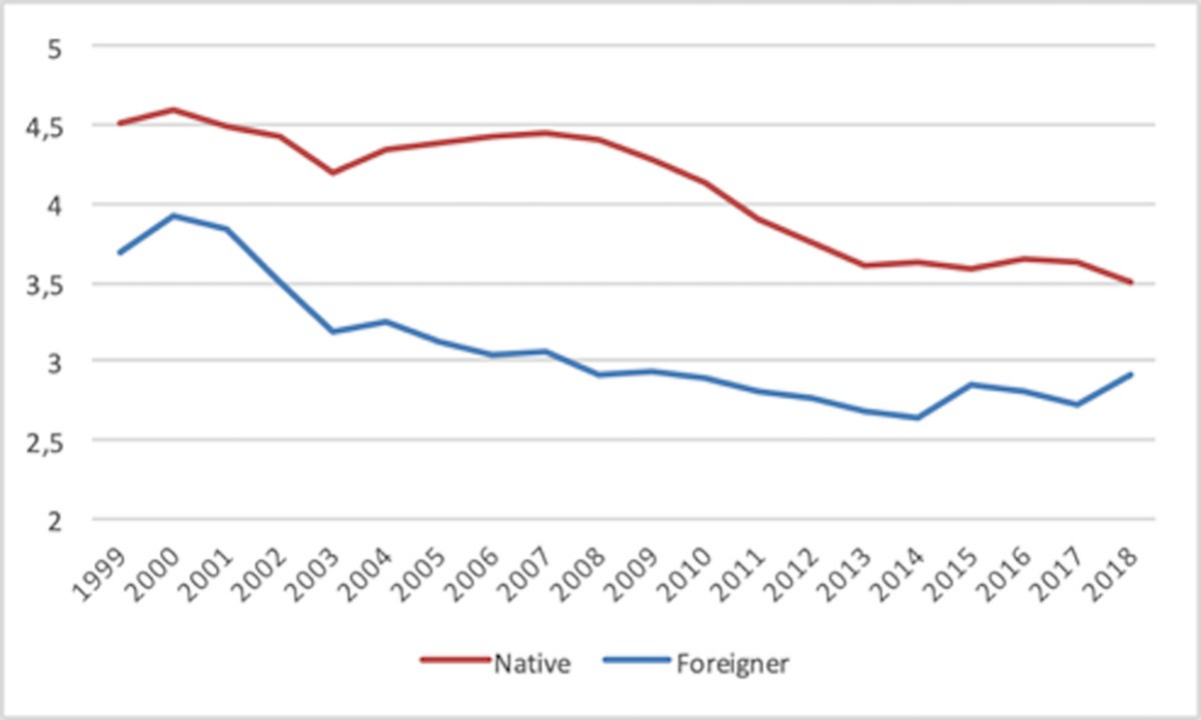
Self-employment rate (Employers) 1999–2018. Country average.

**Fig 4:**
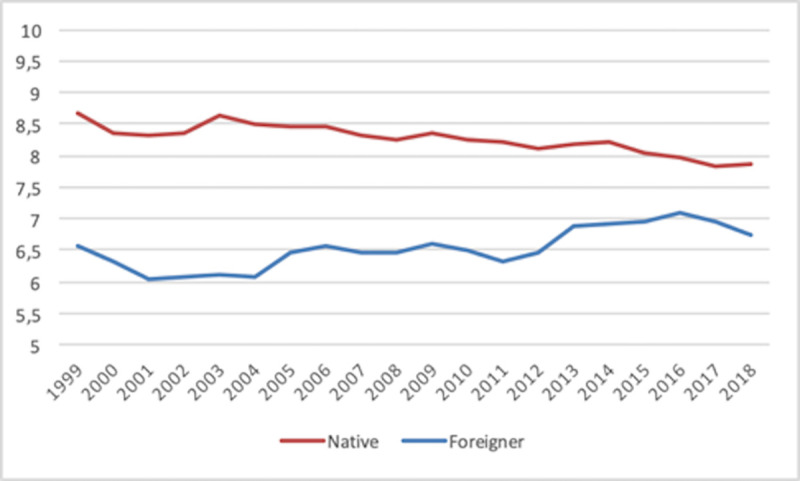
Self-employment rate (Own-account workers) 1999–2018. Country average.

The distinction between employers and own-account workers depicted in Figs 3 and 4 reveals different patterns for the two types of SE. Thus, the rate of employers steadily decreases for both native and foreign-born individuals, although following a slight change of trend in the latter case the rates approach each other on average from 2017 on. In the case of own-account workers, the natives’ average rate is generally fairly steady with a slightly downward trend, whereas the foreign-born rate alternates between a flat trend and (short) upwards periods. As a consequence, both rates have approached each other over time, although a change in trend has appeared since 2017. Obviously, these dynamics correspond to averages, whereas an analysis by country should reveal different geographical realities.

**Fig 5:**
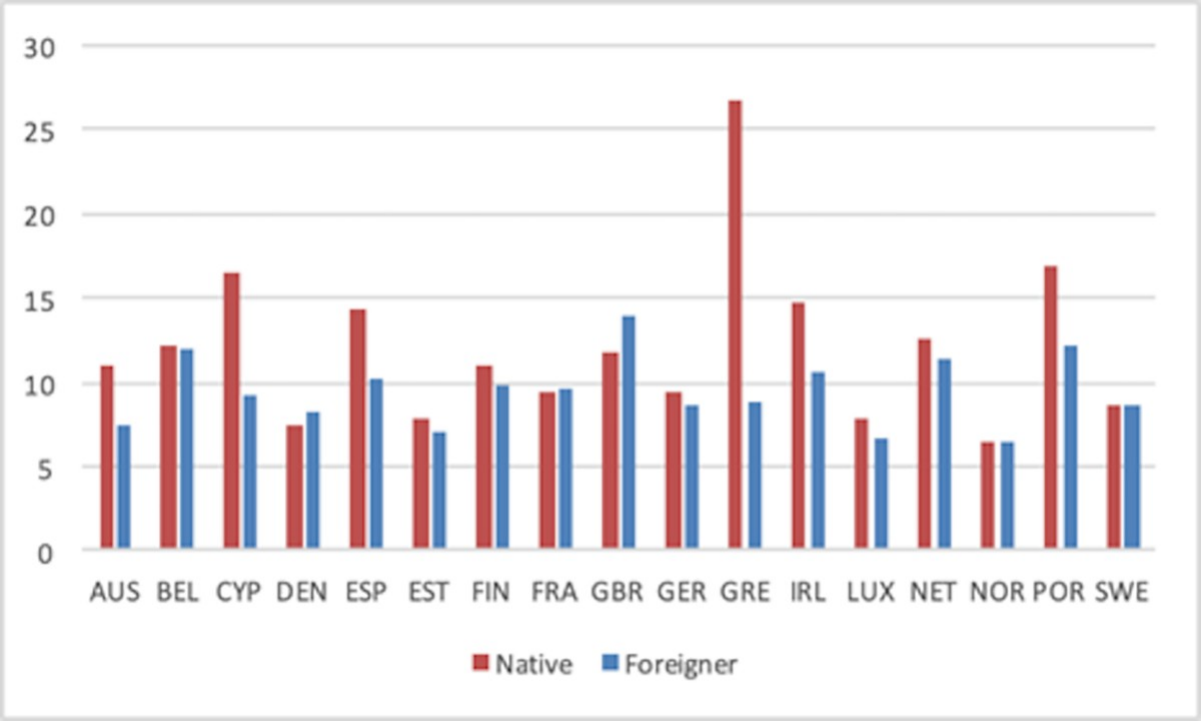
Total self-employment rate by countries (1999–2018 average).

The following figures display SE rates as averages over the whole period considered for each country in our sample. Data by country allow us to confirm that native SE is higher than migrant SE in most countries in our sample (see Fig 5). Greece represents the most extreme case, although those differences are consistently higher for the other Southern European countries (Spain, Portugal, Italy and Cyprus) plus Ireland than for Western European countries such as France or Germany. France plus Northern European and Scandinavian countries display more similar rates for native and foreign-born self-employed (Norway, Sweden, Finland and Estonia) or even slightly higher rates for the latter (Denmark and the UK).

**Fig 6:**
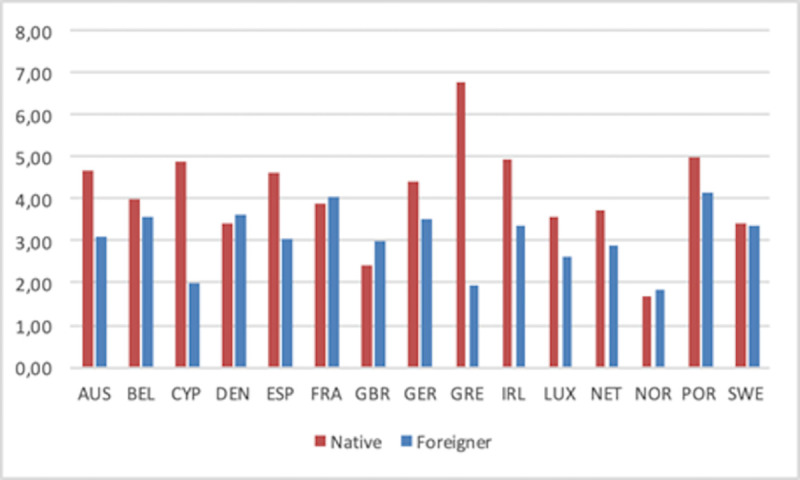
Self-employment rate (Employers) by countries (1999–2018 average).

**Fig 7:**
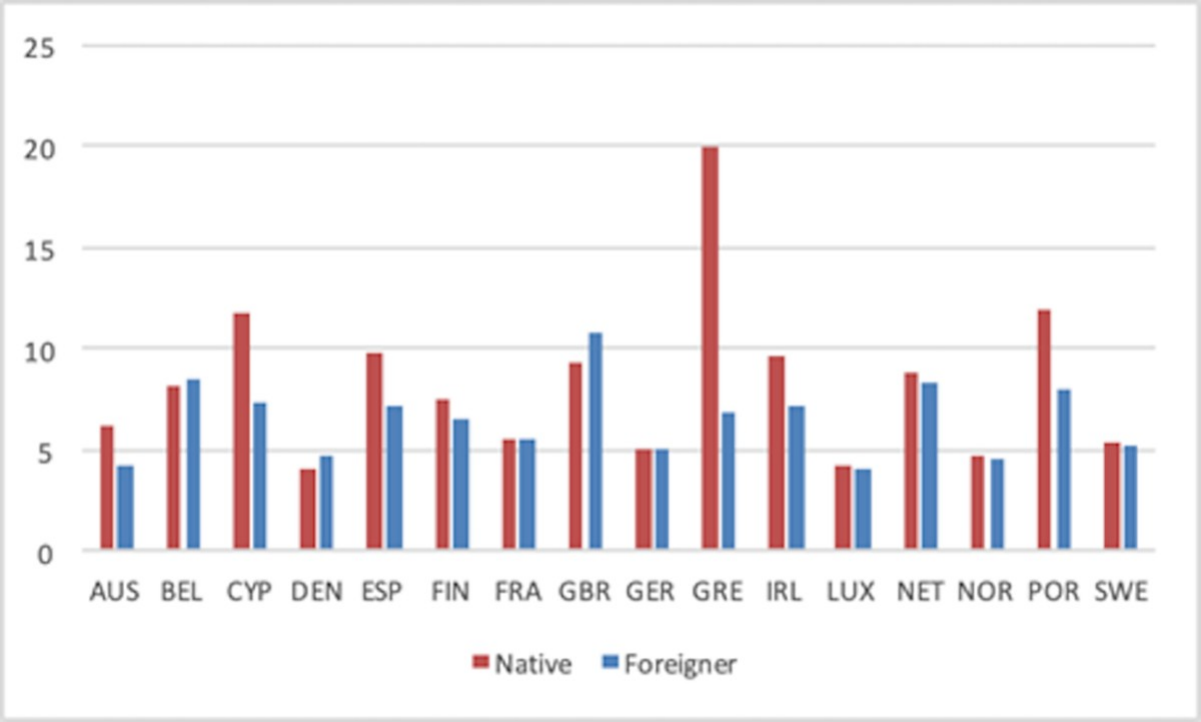
Self-employment rate (Own-account workers) by countries (1999–2018 average).

Disentangling total SE into employers and own-account workers (Figs 6 and 7, respectively) shows that the previous national patterns hold for both types of SE. Thus, native and foreign-born SE profiles by country are very similar in Figs 6 and 7. The main difference is actually the value of the rates, with the former type of SE being systematically smaller than the latter. For readers interested in the profile of each country in all rates over time, we have included S1–S3 Appendices with individual figures for each country.

Now, we analyse whether the previous trends in native and foreign-born SE rates reveal a pattern of convergence. As we stated before, for this analysis we also decompose the aggregate rate into (a) employers and (b) own-account workers.

## 4. Convergence analysis

### 4.1 Econometric methods

To formally test for convergence, we must first define what is understood as convergence. We follow the concepts of sigma convergence (SC) proposed by [33] and long-run convergence by [34].

The former authors define convergence as evidence of a decrease in the gap between income per capita, such that:

$$Y_{t}=\sigma Y_{t-1}+\varepsilon_{t},\;\mathrm{with}\;\sigma <1$$ (1)

where *Y_t_* is the differential between the income per capita in two countries.

Similarly, the definition of [34] implies that converge occurs if the long-term forecasts of the difference in income per capita between two countries are equal at a fixed time, conditional on a set of available information *ϑ*, or in mathematical terms:

$$\sum_{k\to \infty }limE(Y_{t+k}|\vartheta_{t})=0$$ (2)

In both cases the statistical approach may rely on the application of unit root tests or cointegration techniques. Hence, there will be evidence of convergence when the differentials between two variables are stationary.

Equivalently, to test for convergence between SE rates, the following equation is used:

$${se}_{it}=c_{i}+{\theta \hat{se}}_{t}+\varepsilon_{it}$$ (3)

where *se_it_* is the SE rate for country *i* in period *t* and ${\hat{se}}_{t}$ is the average of SE rates for all countries or a value of reference. Testing for absolute convergence would imply *c_i_* = 0 and *θ* = 1, whereas conditional convergence *c_i_* ≠ 0 and *θ* = 1 [12].

In practice, testing for convergence, whether absolute or conditional, involves testing for unit roots in the differential between SE rates defined as $y_{it}={se}_{it}-{\hat{se}}_{t}$.

In this paper we apply unit root tests for panel data, given that the time dimension is not too large and we have a number of countries in the panel. The first test we apply is the one proposed by [35] (LLC), who assume that there is a common unit root process as follows:

$$\Delta y_{it}=\alpha y_{it-1}+\sum_{j=1}^{p_{i}}\beta_{ij}\Delta y_{it-j}+{X\prime }_{it}\delta +\varepsilon_{it}$$ (4)

with *H_0_:α = 0* and *H_1_:α<0*, and *X*′_*it*_ contains fixed effects. This means that under the null there is a unit root whereas under the alternative there is no unit root.

In order to complement our analysis, we also apply the [36] (IPS), the Fisher-Augmented-Dickey-Fuller and Fisher-Phillips-Perron tests, which allow for individual unit root processes. In this case we have:

$$\Delta y_{it}=\alpha_{i}y_{it-1}+\sum_{j=1}^{p_{i}}\beta_{ij}\Delta y_{it-j}+{X\prime }_{it}\delta +\varepsilon_{it}$$ (5)

with *H_0_:α_i_ = 0* and *H_1_:α_j_<0* for *j < i* meaning that under the alternative some individuals are not unit root processes.

Finally, to account for the possibility of cross-sectional dependence we also apply the [37] cross-sectional dependence corrected IPS. This test includes the average of the variable of interest and the lags of its first differences on the right-hand side of the equation:

$$\Delta y_{it}=\alpha_{i}y_{it-1}+\sum_{j=1}^{p_{i}}\beta_{ij}\Delta y_{it-j}+\rho_{i}{\bar{y}}_{it-1}+\sum_{j=1}^{p_{i}}\theta_{ij}\Delta {\bar{y}}_{it-j}+{X\prime }_{it}\delta +\varepsilon_{it}$$ (6)

Since all unit root tests contain fixed effects, we are effectively testing for conditional convergence.

In addition, it is well known that linear unit root tests which do not account for structural breaks may suffer from power problems. However, given the short time dimension of the panel, this feature has not been explored in this paper.

### 4.2 Results

#### 4.2.1 Foreign-born self-employment

We start by analysing the panel unit root tests for the difference between the foreign-born SE rate and the average rate for all countries. The outcome is displayed in column (1) in Table 1. Since we reject the null, this means that there is evidence of convergence towards the average.

**Table 1:** Panel unit root test result foreign vs average.

Method	Total SE (1)	Employers (2)	Own-account workers (3)
Null: Unit root (assumes common unit root process)			
Levin, Lin & Chu t	-2.95799 (<0.01)	-2.66280 (<0.01)	-1.66881 (<0.05)
Null: Unit root (assumes individual unit root process)			
Im, Pesaran and Shin W-stat	-2.29806 (<0.05)	-2.62079 (<0.05)	-1.64627 (<0.05)
Cross-sectional dependence IPS	-2.27016 (<0.05)	-1.80540 (>0.10)	-2.21758 (<0.10)
ADF—Fisher Chi-square	53.1828 (<0.05)	51.7374 (<0.01)	50.6221 (<0.05)
PP—Fisher Chi-square	66.2571 (<0.01)	67.2907 (<0.01)	59.1321 (<0.01)
Cross-sections	17	15(*)	16 (**)

Probabilities in parentheses.

Probabilities for Fisher tests are computed using an asymptotic Chi-square distribution. All other tests assume asymptotic normality

(*) Due to unavailability of data, Estonia and Finland are removed from the sample

(**) Due to unavailability of data, Estonia is removed from the sample

In column (2) we show the results for the differences between the SE rates for employers and the average of the same variable for the countries analysed. Due to a lack of data, Estonia and Finland are not included in the pool. The results point in the same direction as the ones reported in column (1), i.e. there is evidence of convergence, at least in some cases, in the SE rates *vs* the average of all countries.

In column (3) we show the results for the panel unit root tests of the differences between SE rates for own-account workers and the average of all the countries. For these tests, Estonian data are not available and hence the pool does not contain this Baltic country. Once again, we find evidence of convergence with the average.

According to this evidence, foreign self-employed workers, regardless of their countries of origin and the country where they live, converge to a common average. This result holds for both types of SE, although each type obviously converges to a different average. The relevance of this finding is that it suggests that being self-employed in a foreign country leads to shared patterns across countries that outweigh other specific aspects, such as individuals’ own culture or the characteristics of the host country. This interpretation is consistent with empirical research that has proven that self-employed migrants are (for all countries of origin and residence) more responsive to changes in labour markets scenarios than their native counterparts are [38].

The speed of adjustment is related to the *α_i_* in Eqs (4) and (5). The estimated parameters for columns (1), (2) and (3) are -0.18, -0.32 and -0.17, respectively. This means that a one-percent shock disappears at a rate between 17 and 32% per year. The speed of adjustment is faster for employers.

#### 4.2.2 Native self-employment

We repeat the exercise for the difference between the SE rate for natives and the average rate for all countries. The results are shown in Table 2. For the total SE rate (statistics displayed in column (4)), we find that in none of the cases is the null of unit root rejected, implying that there is no evidence of convergence towards the mean. In contrast to what we have found in Table 1, this implies that in none of the countries analysed do the SE rates for natives converge to the average of the 17 countries.

**Table 2:** Panel unit root test result native vs average.

Method	Total SE (4)	Employers (5)	Own-account workers (6)
Null: Unit root (assumes common unit root process)			
Levin, Lin & Chu t	-0.76572 (>0.10)	-1.92717 (<0.05)	-1.94439 (<0.05)
Null: Unit root (assumes individual unit root process)			
Im, Pesaran and Shin W-stat	1.57009 (>0.10)	-0.33783 (>0.10)	-0.08362 (>0.10)
Cross-sectional dependence IPS	-2.39089 (<0.05)	-1.90409 (>0.10)	-1.57171 (>0.10)
ADF—Fisher Chi-square	24.2026 (>0.10)	31.1057 (>0.10)	36.0531 (>0.10)
PP—Fisher Chi-square	19.4462 (>0.10)	25.3758 (>0.10)	37.2099 (>0.10)
Cross-sections	17	15(*)	16(**)

Probabilities in parentheses.

Probabilities for Fisher tests are computed using an asymptotic Chi-square distribution. All other tests assume asymptotic normality

(*) Due to unavailability of data, Estonia and Finland are removed from the sample

(**) Due to unavailability of data, Estonia is removed from the sample

Nevertheless, the LLC tests yield some evidence of convergence for the cases where we separate job creators and SE without employees.

Contrary to the results for foreign-born SE rates, these tests provide less robust evidence, which suggests that it is more difficult to achieve convergence in native SE rates. According to this view, country idiosyncrasies, i.e. cultural particularities, attitudes towards SE, and institutional and legal issues, are likely to prevail over the shared characteristics that lead individuals to choose SE.

The estimated autoregressive parameters in columns (5) and (6), only for the stationary cases, are -0.18 and -0.08, respectively. As before, the speed of convergence is faster for the employers.

#### 4.2.3 Convergence between native and foreign-born self-employment

We now take the difference between native and foreign-born SE rates for each country and apply panel unit root tests. With this analysis, we can assess how the SE rates for foreign-born individuals converge to the SE rates of natives. The results are displayed in Table 3.

**Table 3:** Panel unit root test result native vs foreign.

Method	Total SE (7)	Employers (8)	Own-account workers (9)
Null: Unit root (assumes common unit root process)			
Levin, Lin & Chu t	0.07832 (>0.10)	-3.25064 (<0.05)	-3.83441 (<0.05)
Null: Unit root (assumes individual unit root process)			
Im, Pesaran and Shin W-stat	-0.98071 (>0.10)	-2.81935 (<0.01)	-2.95005 (<0.01)
Cross-sectional dependence IPS	-2.52981 (<0.01)	-2.74171 (<0.01)	-2.77873 (<0.01)
ADF—Fisher Chi-square	47.1631 (<0.10)	65.7118 (<0.01)	65.7279 (<0.01)
PP—Fisher Chi-square	57.7944 (<0.01)	77.5268 (<0.01)	58.3450 (<0.01)
Cross-sections	17	15(*)	16(**)

Probabilities in parentheses.

Probabilities for Fisher tests are computed using an asymptotic Chi-square distribution. All other tests assume asymptotic normality

(*) Due to unavailability of data, Estonia and Finland are removed from the sample

(**) Due to unavailability of data, Estonia is removed from the sample

According to the results displayed in column (7), only in the case of the PP test are we able to reject the null. This means that there is very little evidence in favour of the hypothesis that the total SE rates of native and foreign-born individuals converge to one another within our target countries.

If we take the difference between the rates of SE for employer, we find, as depicted in column (8), that there is evidence of intra-country convergence. This contrasts with the results obtained for the total SE rate. Finally, we present in column (9) the results of the unit root test for the difference between native and foreign-born rates of own-account workers. The results are similar to those of column (8).

This means that when it comes to SE for employers and own-account workers separately, the evolution of the rates seems to be similar between the native and foreign-born population. These results may seem counterintuitive since for the total SE we cannot reject the null of unit root, implying no convergence. The reason is that the two subgroups, employers and own-account workers, converge to different means: 1% and 1.73%, respectively.

The autoregressive parameters for columns (8) and (9), the stationary cases, are -0.32 and -0.25, respectively, showing a fairly fast mean reversion. As before, employers tend to converge faster.

In summary, we can say that it seems that foreign-born workers’ behaviour is more similar across countries than native’s workers’ behaviour when comparing between countries. In addition, while we find no evidence of convergence when we compare foreign-born and native workers for the overall SE rates, we do find some evidence of convergence when we distinguish between employers and own-account workers.

## 5. Conclusions

Research on the convergence in self-employment (SE) rates has been gathering momentum, as it is a good way to assess real convergence and whether the EU policies are having an impact on the SE rates. This paper examines the patterns of convergence in the SE rates for both foreign-born and native populations in a sample of 17 European countries. We find that foreign-born SE rates converge to the average of all countries analysed in the three classifications that we have used (total SE, employers and own-account workers). However, the evidence for the convergence of native SE rates is rather weak. This outcome could suggest that foreign-born self-employed workers share common characteristics across countries (specifically, the fact that they are migrants) whereas natives are more sensitive to local conditions. When looking at the convergence of native towards foreign-born SE, we find that there is evidence of convergence only when analysing employers and own-account workers separately. As mentioned above, this may be due to the fact that the two groups convergence to different means.

These results point in three directions, which we consider relevant in the European context, where the SE rate of migrants is lower than that of natives. First, policy measures aimed at fostering the integration of migrants could help the foreign-born population to ease this convergence towards the (usually) higher SE rates of natives, while also helping the necessity self-employed. Second, and related to the first issue, is it not entirely obvious from our results that specific policies to foment migrant SE are required, rather than simply encouraging all individuals to participate in SE. In other words, we should be careful about putting too much emphasis on promoting migrant SE rather than facilitating migrants’ access to labour markets and the formal economy on par with native workers.

Finally, our findings imply that foreign-born workers tend to lead to a homogenisation of SE rates among the countries analysed. Indeed, it seems that they behave more homogeneously than the native populations when comparing between the different countries. We are not implying that they boost the SE rates, something we do not analyse in this paper, but the fact that they behave in a similar manner may help strengthen EU policies. In other words, the fact that non-native self-employed behave similarly across countries makes EU policies easier to implement.

## Supporting information

S1 Appendix Total SE over active population (%). A1-1 to A1-15 Figs.
(ZIP)

S2 Appendix Employers. Total SE over active population (%). A2-1 to A2-15 Figs.
(ZIP)

S3 Appendix Own-account workers. Total SE over active population (%). A3-1 to A3-16 Figs.
(ZIP)
