# Supplementary figures and images for: Self-employment convergence in Europe: The role of migration
Source: PLoS One. 2021 Apr 22;16(4):e0250182. doi: 10.1371/journal.pone.0250182 (PMC8062057; doi:10.1371/journal.pone.0250182)

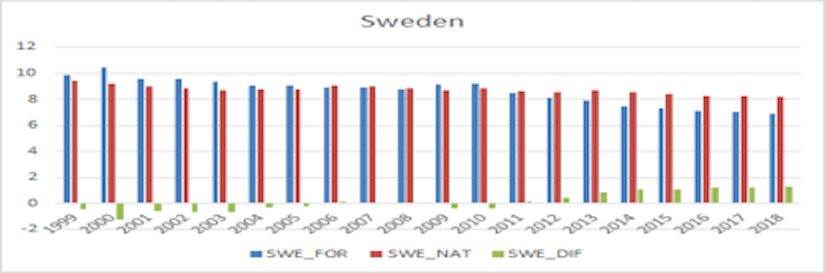

Supplement: S1 Appendix — A1-1 to A1-15 Figs. (ZIP) [file pone.0250182.s001.zip › Figure A1-17.tif]

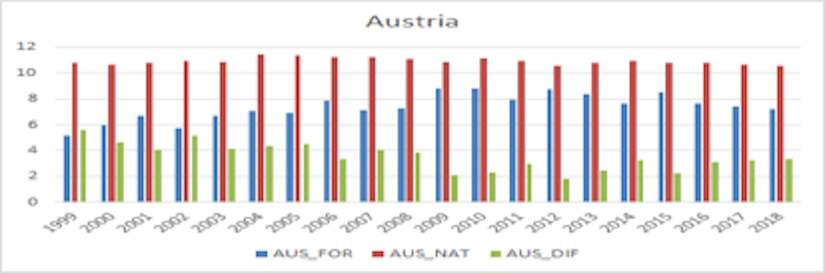

Supplement: S1 Appendix — A1-1 to A1-15 Figs. (ZIP) [file pone.0250182.s001.zip › Figure A1-1.tif]

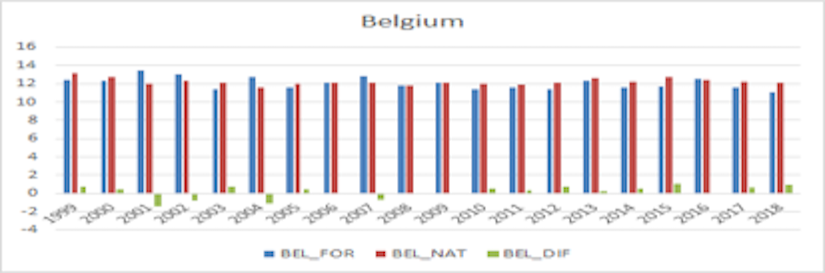

Supplement: S1 Appendix — A1-1 to A1-15 Figs. (ZIP) [file pone.0250182.s001.zip › Figure A1-2.tif]

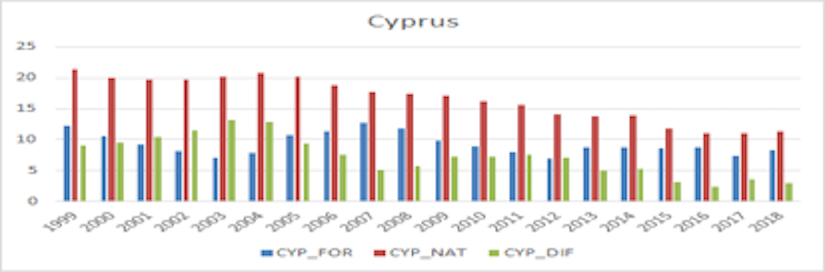

Supplement: S1 Appendix — A1-1 to A1-15 Figs. (ZIP) [file pone.0250182.s001.zip › Figure A1-3.tif]

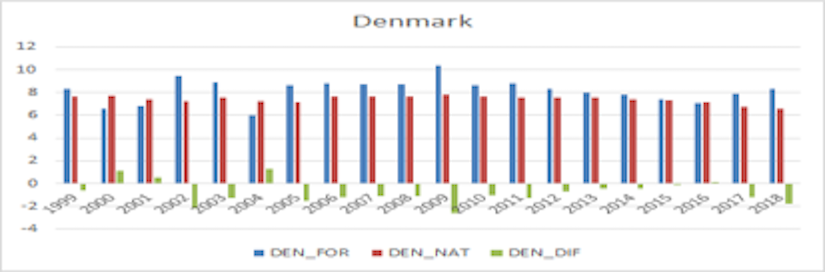

Supplement: S1 Appendix — A1-1 to A1-15 Figs. (ZIP) [file pone.0250182.s001.zip › Figure A1-4.tif]

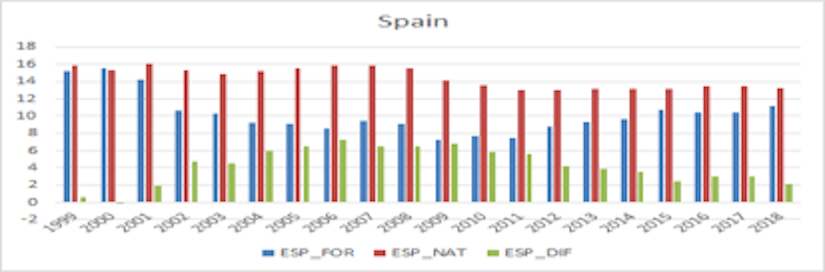

Supplement: S1 Appendix — A1-1 to A1-15 Figs. (ZIP) [file pone.0250182.s001.zip › Figure A1-5.tif]

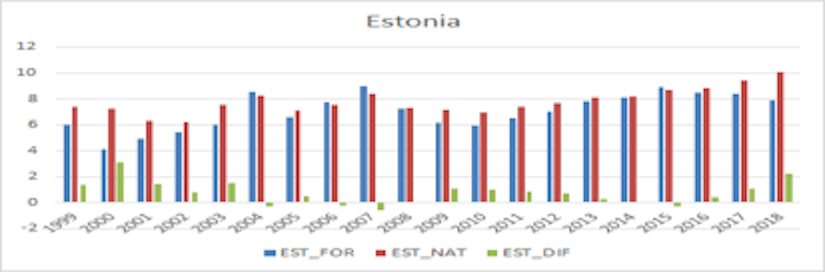

Supplement: S1 Appendix — A1-1 to A1-15 Figs. (ZIP) [file pone.0250182.s001.zip › Figure A1-6.tif]

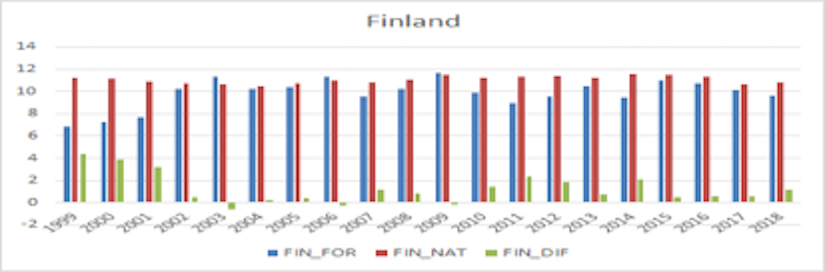

Supplement: S1 Appendix — A1-1 to A1-15 Figs. (ZIP) [file pone.0250182.s001.zip › Figure A1-7.tif]

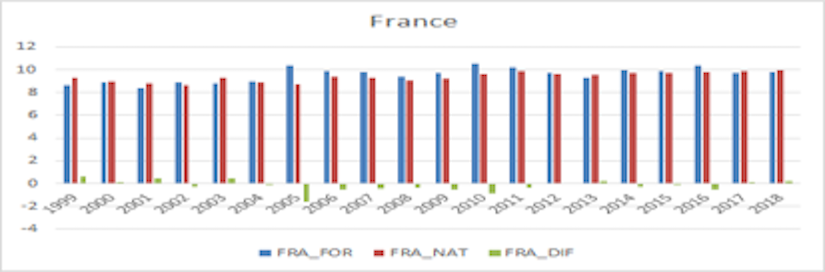

Supplement: S1 Appendix — A1-1 to A1-15 Figs. (ZIP) [file pone.0250182.s001.zip › Figure A1-8.tif]

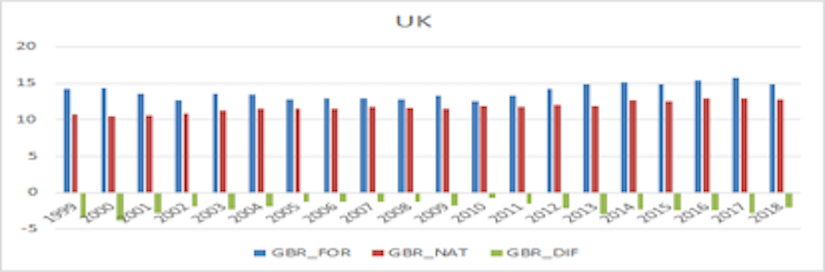

Supplement: S1 Appendix — A1-1 to A1-15 Figs. (ZIP) [file pone.0250182.s001.zip › Figure A1-9.tif]

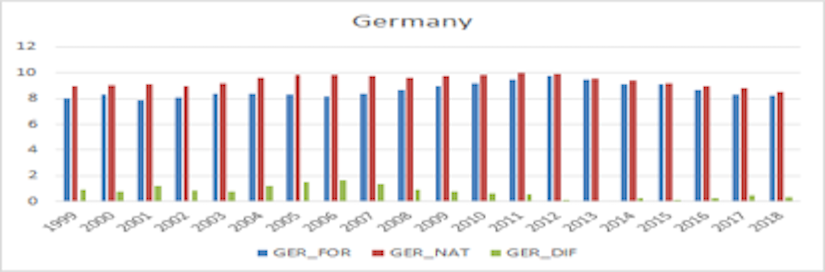

Supplement: S1 Appendix — A1-1 to A1-15 Figs. (ZIP) [file pone.0250182.s001.zip › Figure A1-10.tif]

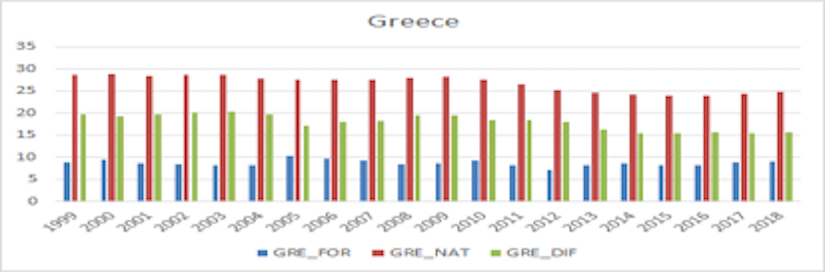

Supplement: S1 Appendix — A1-1 to A1-15 Figs. (ZIP) [file pone.0250182.s001.zip › Figure A1-11.tif]

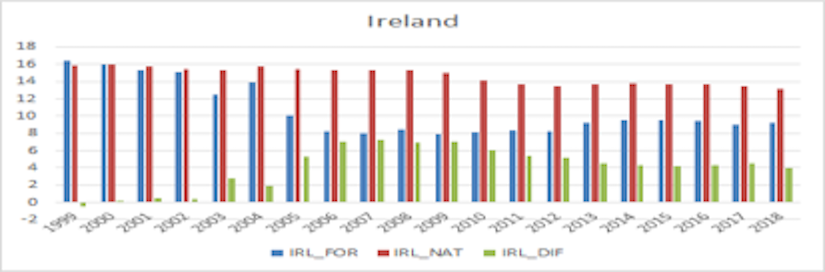

Supplement: S1 Appendix — A1-1 to A1-15 Figs. (ZIP) [file pone.0250182.s001.zip › Figure A1-12.tif]

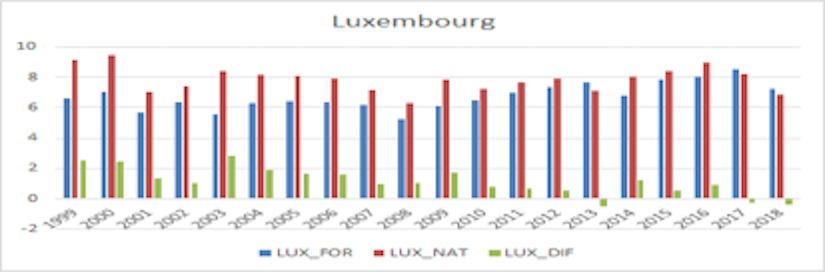

Supplement: S1 Appendix — A1-1 to A1-15 Figs. (ZIP) [file pone.0250182.s001.zip › Figure A1-13.tif]

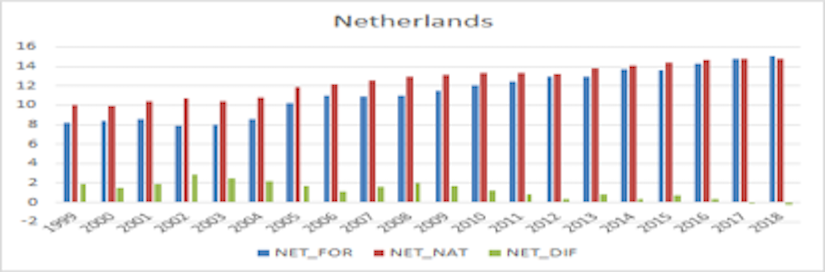

Supplement: S1 Appendix — A1-1 to A1-15 Figs. (ZIP) [file pone.0250182.s001.zip › Figure A1-14.tif]

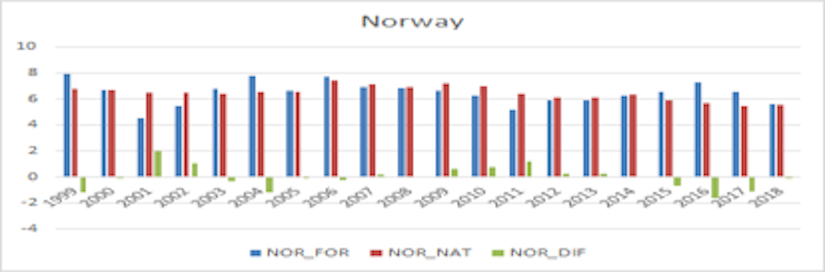

Supplement: S1 Appendix — A1-1 to A1-15 Figs. (ZIP) [file pone.0250182.s001.zip › Figure A1-15.tif]

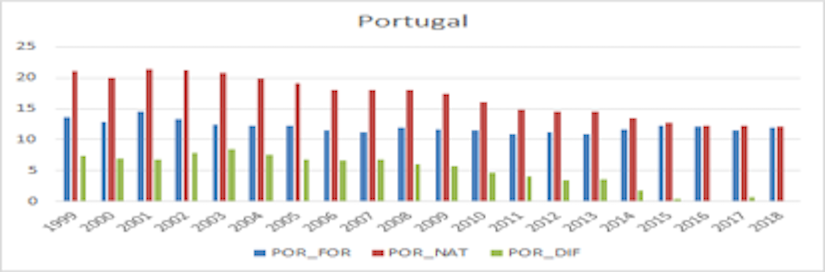

Supplement: S1 Appendix — A1-1 to A1-15 Figs. (ZIP) [file pone.0250182.s001.zip › Figure A1-16.tif]

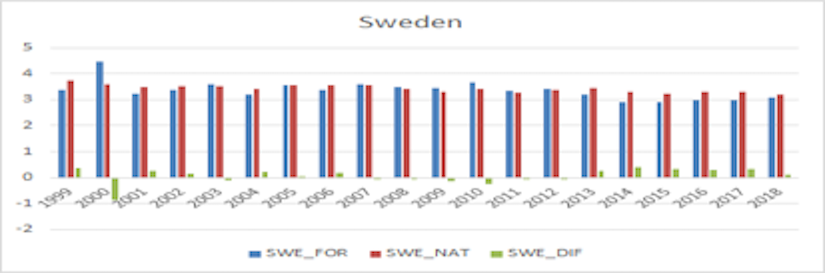

Supplement: S2 Appendix — Total SE over active population (%). A2-1 to A2-15 Figs. (ZIP) [file pone.0250182.s002.zip › FIgure A2-15.tif]

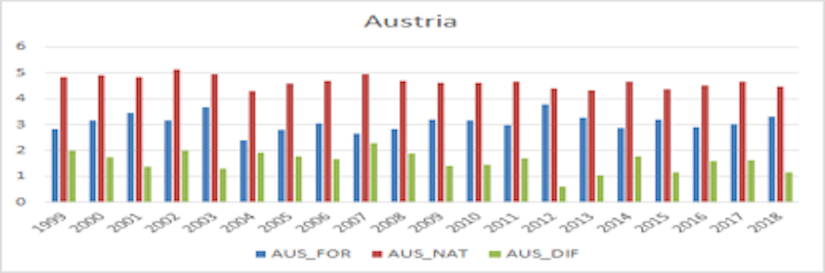

Supplement: S2 Appendix — Total SE over active population (%). A2-1 to A2-15 Figs. (ZIP) [file pone.0250182.s002.zip › Figure A2-1.tif]

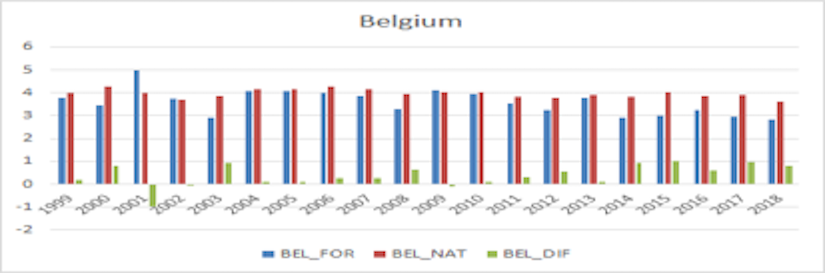

Supplement: S2 Appendix — Total SE over active population (%). A2-1 to A2-15 Figs. (ZIP) [file pone.0250182.s002.zip › Figure A2-2.tif]

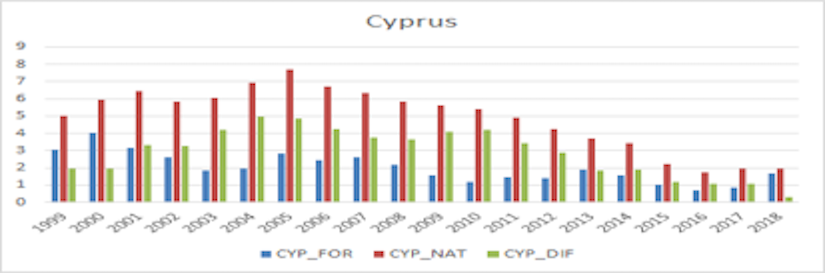

Supplement: S2 Appendix — Total SE over active population (%). A2-1 to A2-15 Figs. (ZIP) [file pone.0250182.s002.zip › Figure A2-3.tif]

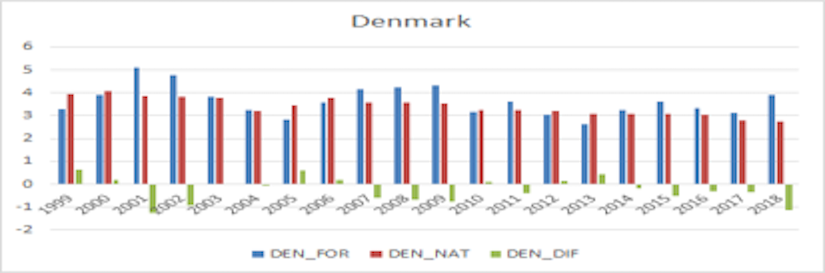

Supplement: S2 Appendix — Total SE over active population (%). A2-1 to A2-15 Figs. (ZIP) [file pone.0250182.s002.zip › Figure A2-4.tif]

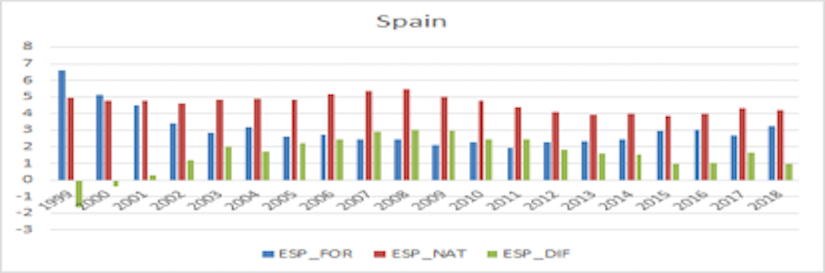

Supplement: S2 Appendix — Total SE over active population (%). A2-1 to A2-15 Figs. (ZIP) [file pone.0250182.s002.zip › Figure A2-5.tif]

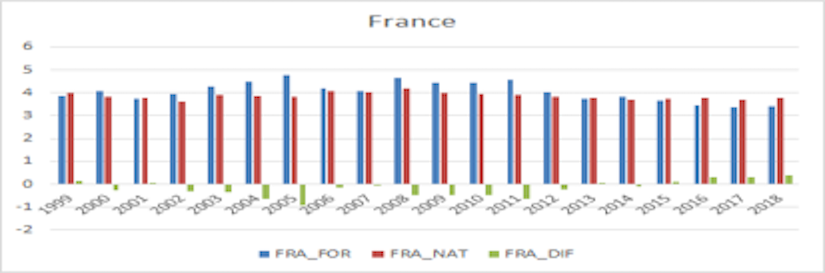

Supplement: S2 Appendix — Total SE over active population (%). A2-1 to A2-15 Figs. (ZIP) [file pone.0250182.s002.zip › Figure A2-6.tif]

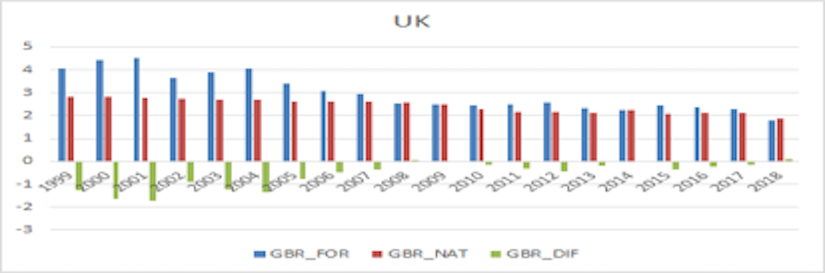

Supplement: S2 Appendix — Total SE over active population (%). A2-1 to A2-15 Figs. (ZIP) [file pone.0250182.s002.zip › Figure A2-7.tif]

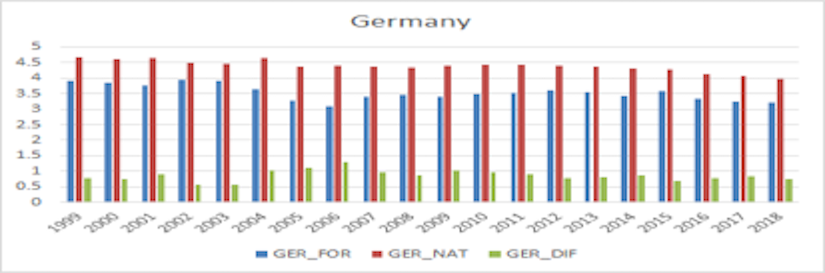

Supplement: S2 Appendix — Total SE over active population (%). A2-1 to A2-15 Figs. (ZIP) [file pone.0250182.s002.zip › Figure A2-8.tif]

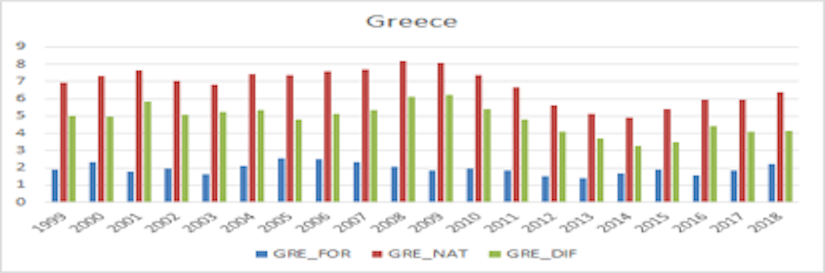

Supplement: S2 Appendix — Total SE over active population (%). A2-1 to A2-15 Figs. (ZIP) [file pone.0250182.s002.zip › Figure A2-9.tif]

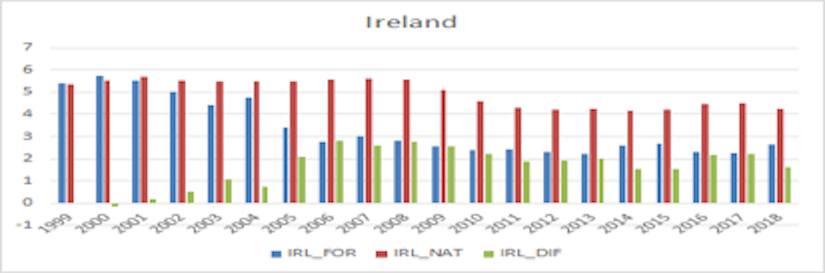

Supplement: S2 Appendix — Total SE over active population (%). A2-1 to A2-15 Figs. (ZIP) [file pone.0250182.s002.zip › Figure A2-10.tif]

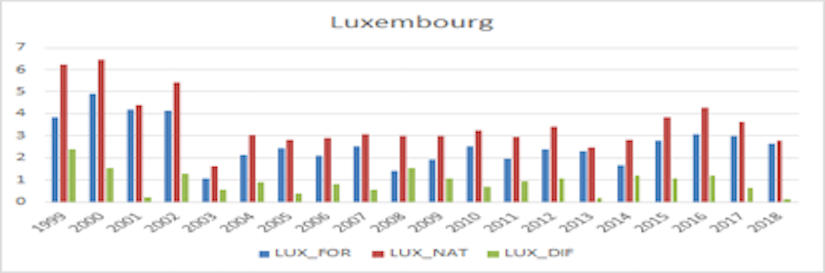

Supplement: S2 Appendix — Total SE over active population (%). A2-1 to A2-15 Figs. (ZIP) [file pone.0250182.s002.zip › Figure A2-11.tif]

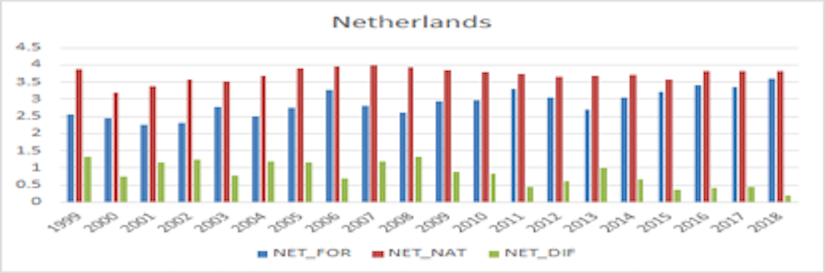

Supplement: S2 Appendix — Total SE over active population (%). A2-1 to A2-15 Figs. (ZIP) [file pone.0250182.s002.zip › Figure A2-12.tif]

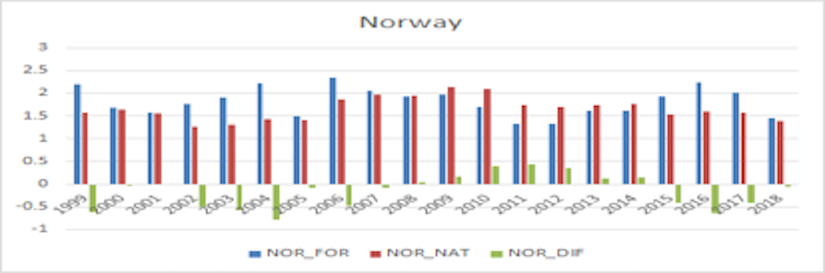

Supplement: S2 Appendix — Total SE over active population (%). A2-1 to A2-15 Figs. (ZIP) [file pone.0250182.s002.zip › Figure A2-13.tif]

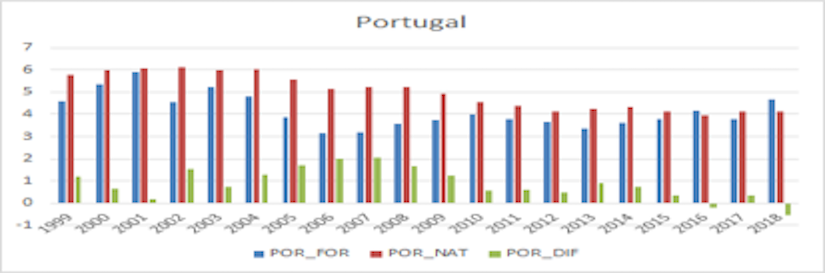

Supplement: S2 Appendix — Total SE over active population (%). A2-1 to A2-15 Figs. (ZIP) [file pone.0250182.s002.zip › Figure A2-14.tif]

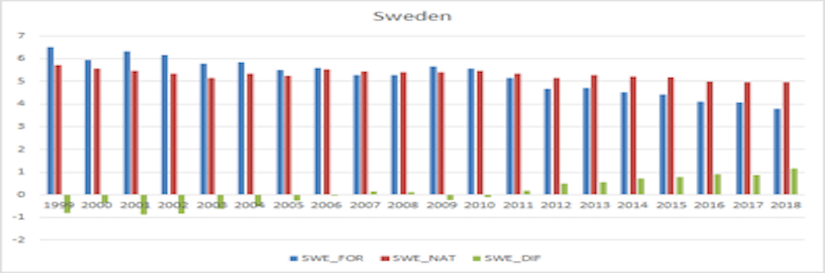

Supplement: S3 Appendix — Total SE over active population (%). A3-1 to A3-16 Figs. (ZIP) [file pone.0250182.s003.zip › Figure A3-16.tif]

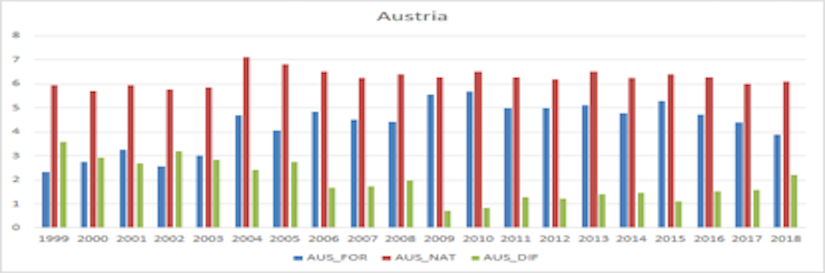

Supplement: S3 Appendix — Total SE over active population (%). A3-1 to A3-16 Figs. (ZIP) [file pone.0250182.s003.zip › Figure A3-1.tif]

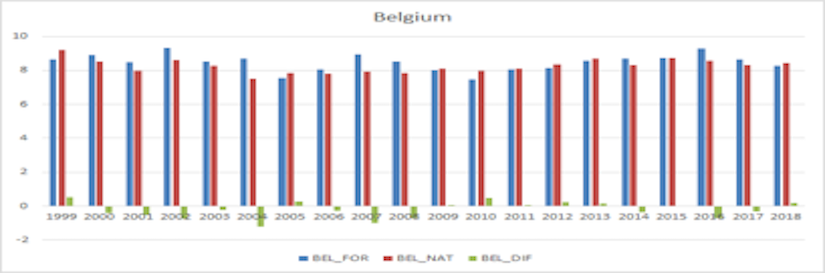

Supplement: S3 Appendix — Total SE over active population (%). A3-1 to A3-16 Figs. (ZIP) [file pone.0250182.s003.zip › Figure A3-2.tif]

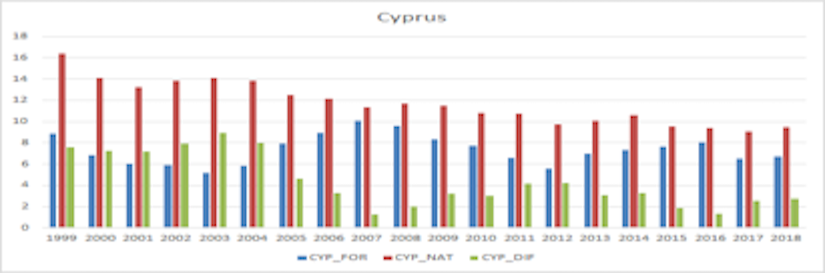

Supplement: S3 Appendix — Total SE over active population (%). A3-1 to A3-16 Figs. (ZIP) [file pone.0250182.s003.zip › Figure A3-3.tif]

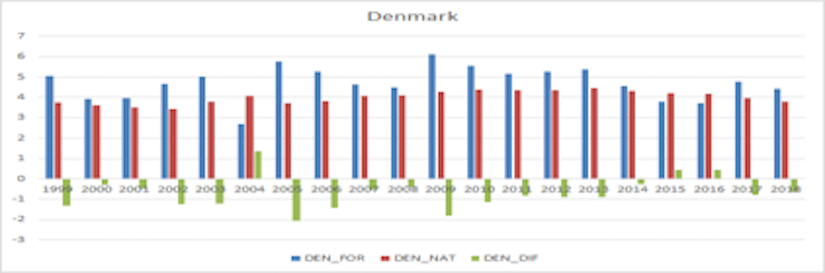

Supplement: S3 Appendix — Total SE over active population (%). A3-1 to A3-16 Figs. (ZIP) [file pone.0250182.s003.zip › Figure A3-4.tif]

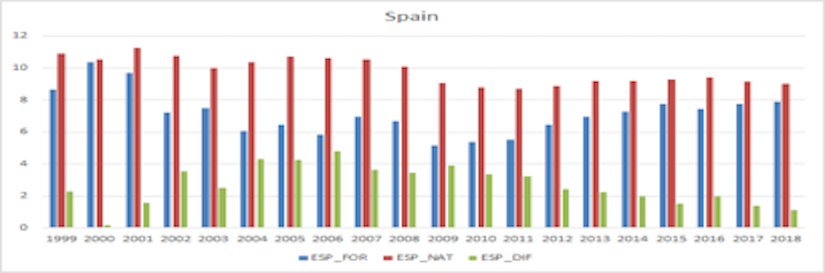

Supplement: S3 Appendix — Total SE over active population (%). A3-1 to A3-16 Figs. (ZIP) [file pone.0250182.s003.zip › Figure A3-5.tif]

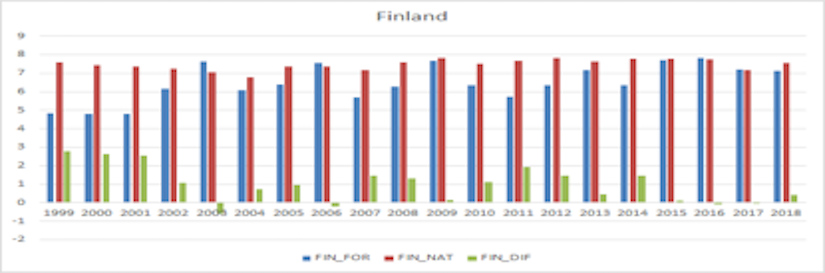

Supplement: S3 Appendix — Total SE over active population (%). A3-1 to A3-16 Figs. (ZIP) [file pone.0250182.s003.zip › Figure A3-6.tif]

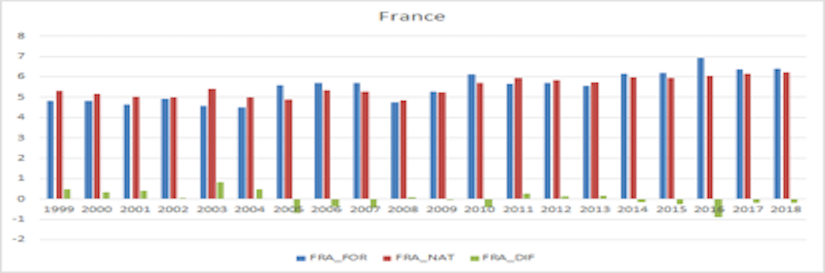

Supplement: S3 Appendix — Total SE over active population (%). A3-1 to A3-16 Figs. (ZIP) [file pone.0250182.s003.zip › Figure A3-7.tif]

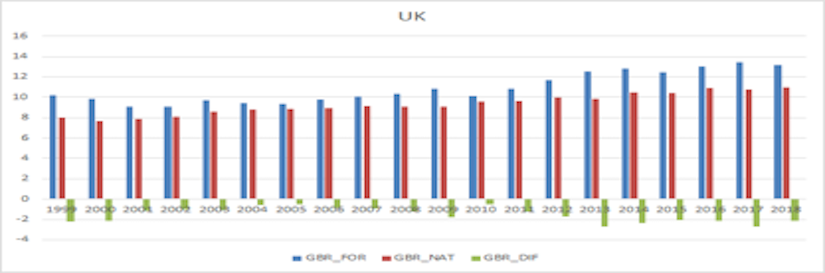

Supplement: S3 Appendix — Total SE over active population (%). A3-1 to A3-16 Figs. (ZIP) [file pone.0250182.s003.zip › Figure A3-8.tif]

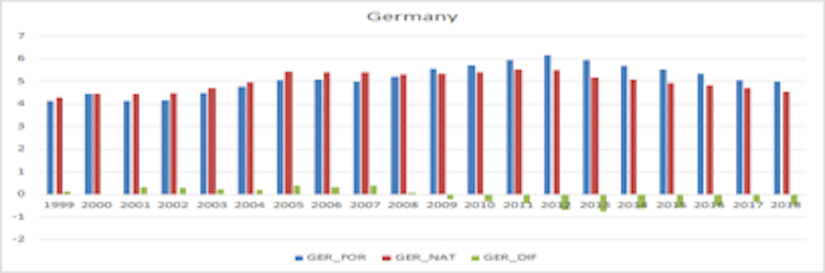

Supplement: S3 Appendix — Total SE over active population (%). A3-1 to A3-16 Figs. (ZIP) [file pone.0250182.s003.zip › Figure A3-9.tif]

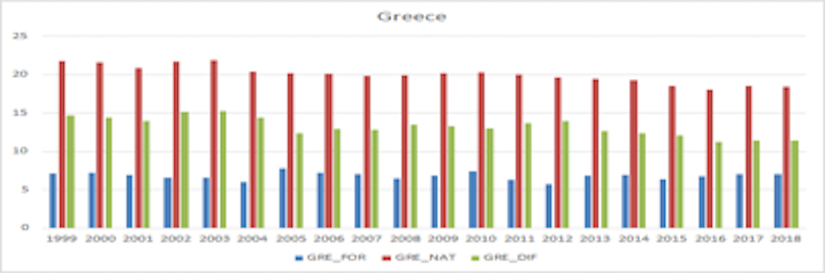

Supplement: S3 Appendix — Total SE over active population (%). A3-1 to A3-16 Figs. (ZIP) [file pone.0250182.s003.zip › Figure A3-10.tif]

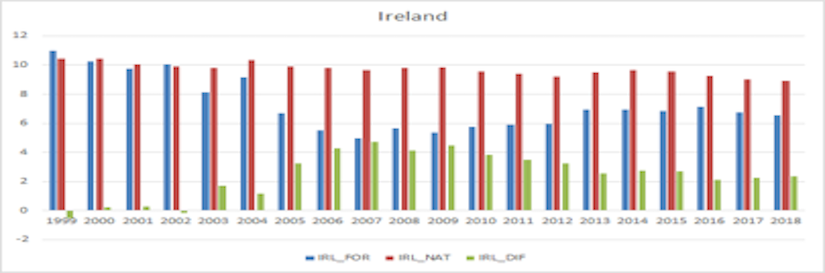

Supplement: S3 Appendix — Total SE over active population (%). A3-1 to A3-16 Figs. (ZIP) [file pone.0250182.s003.zip › Figure A3-11.tif]

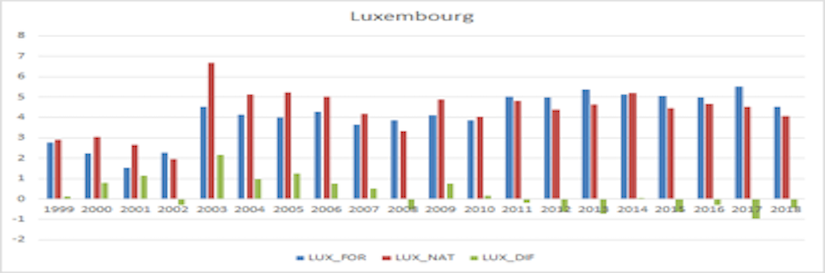

Supplement: S3 Appendix — Total SE over active population (%). A3-1 to A3-16 Figs. (ZIP) [file pone.0250182.s003.zip › Figure A3-12.tif]

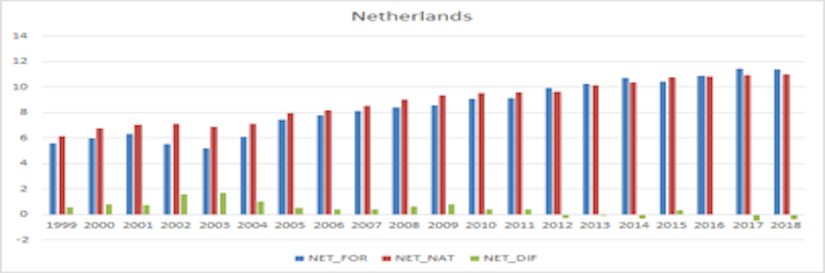

Supplement: S3 Appendix — Total SE over active population (%). A3-1 to A3-16 Figs. (ZIP) [file pone.0250182.s003.zip › Figure A3-13.tif]

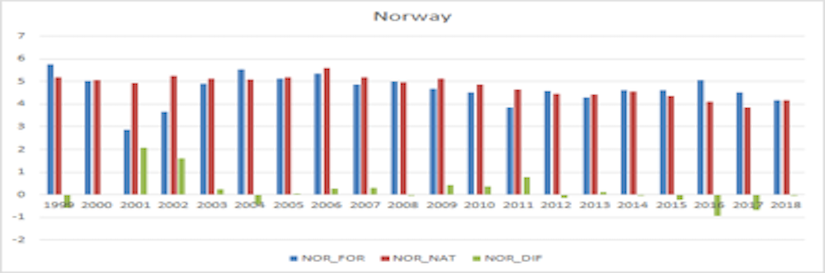

Supplement: S3 Appendix — Total SE over active population (%). A3-1 to A3-16 Figs. (ZIP) [file pone.0250182.s003.zip › Figure A3-14.tif]

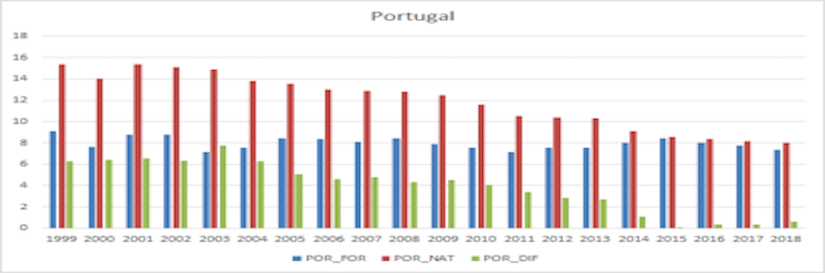

Supplement: S3 Appendix — Total SE over active population (%). A3-1 to A3-16 Figs. (ZIP) [file pone.0250182.s003.zip › Figure A3-15.tif]
